# Haemostatics in surgery and our experience in the enucleoresection of renal cell carcinoma

**DOI:** 10.1186/1477-7819-8-37

**Published:** 2010-05-12

**Authors:** Gianna Pace, Pietro Saldutto, Carlo Vicentini, Lucio Miano

**Affiliations:** 1 Department of Surgical Sciences, University of L'Aquila, San Salvatore Street, Palace 6 A, Coppito, 67100 L'Aquila, Italy; 2 Department of Health Sciences, University of L'Aquila, San Salvatore Street, Palace 6 A, Coppito, 67100 L'Aquila, Italy; 3 Department of Urology, Mazzini Hospital, Italy Square, Teramo, Italy; 4 Department of Urology, Sant'Andrea Hospital, University La Sapienza, Rome, Italy

## Abstract

**Background:**

30 patients, with T1 renal cell carcinomas (RCC) who underwent open enucleoresection of the tumour, were randomized to the use of a topical haemostatic agent (Floseal) or to an infrared-sapphire coagulator (ISC), to compare their efficacy in achieving haemostasis. Methods: Successful intra-operative haemostasis, intra- and post-operative bleeding, operative time, hospital discharge were evaluated.

**Results:**

Statistically higher rates of successful haemostasis and shorter time-to-haemostasis (8,1 vs 12,9 min) were observed in the FloSeal group (p < 0.001 both). Patients operative time was not different between Group 1 vs 2 (58.7 ± 12 vs 62.4 ± 15; p > 0.05). The average blood loss during surgery was less (60 +/- 25.5 mL) for the FloSeal group than for the ISC group (85 +/- 40.5 mL) (p < 0.05). Postoperative blood loss was 25 +/- 5 mL and 40 +/- 45 mL for Floseal and ISC respectively, (p < 0.05). Length of the postoperative hospital discharge was 2.5 +/- 1.2 days for FloSeal group and 3.5 +/- 1.3 for the Group 2 (p < 0.05). No major immediate or delayed complications were observed in either Groups.

**Conclusions:**

The use of Floseal and ISC offer a safe and efficacy haemostasis in the enucleoresection of RCC. Moreover, our results show a less intra-operative and post-operative blood loss as well as a shorter time to haemostasis of Floseal in respect to ISC.

## Background

As the number of minimally invasive and laparoscopic procedures increases, haemostatic agents (HAs) are becoming more popular as a means of achieving rapid haemostasis. Although the recently widespread acceptance, confusion still persists about their indications for use and the optimal agent choice. They comprise a wide range of components including topical hemostats, anti-fibrinolytics, fibrin sealants and matrix hemostats. Topical HAs composed of a gelatin-based matrix and thrombin have been reported to be effective, in addition to traditional means, in terminating bleeding during cardiac operations in comparison with haemostatic patches or sponges composed of either oxidized regenerated cellulose or purified porcine skin gelatin [1]. The haemostatic efficacy and handling of gelatin-thrombin matrix has been proven also in the uterine bleeding, during abdominal myomectomy and, in thyroid surgery [2-5]. Adequate haemostasis is extremely important in neurosurgery. In patients with supratentorial intracerebral hematomas FloSeal, injected into the surgical cavity, has reduced brain exposure, damage to the surrounding tissue and the length of surgery. Furthermore, application of FloSeal at a laminectomy site may be useful to decrease adhesion at the interface between the dura mater and the epidural fibrosis [6,7]. Moreover, the management of intradural bleeding during extended endoscopic endonasal surgery has been challenged by applying a thrombin-gelatin haemostatic matrix, useful for both oozing and focal hemorrhage and effective even for high-flow bleeding [8]. Recently, Izzo et al. reported a large prospective study with the use of HAs in patients undergoing major hepatic surgery providing a rapid and effective intra-operative control of mild to severe bleeding from the liver edge [9]. In animal models, comparing safety, efficacy, presence of residual material and foreign body reaction of commonly used agents such as microporous polysaccharide hemospheres (Arista), oxidized cellulose (Surgicel), microfibrillar collagen (Avitene) and gelatin matrix thrombin sealant (FloSeal) emerged that Arista, Avitene, FloSeal, and Surgicel performed better haemostasis; residual material was not present with Arista, contrasting with its presence in 100% of lesions using Avitene, FloSeal, and Surgicel; furthermore Avitene and FloSeal demonstrated a propensity for causing granuloma formation, whereas Arista and Surgicel showed no such evidence. Arista degrades more rapidly than Surgicel, Avitene and FloSeal and it does not result in any foreign body reaction [10].

Focusing on the urologic applications of tissue glues and HAs, they have been used in the management of genitourinary injuries, surgical wounds, and complications. The best evidence for efficacy and safety exists for haemostasis, especially for nephrectomy and trauma. Newer data highlight urinary tract reconstruction, fistula and percutaneous tract closure, suture line strengthening and infertility as potential uses.

Partial nephrectomy (PN) is a procedure frequently reserved for small, peripherally located renal tumours and the intra- and post-operative haemorrhage represents the most significant risk associated to surgery.

Based upon such considerations and as HAs have become increasingly employed across all surgical fields, we aim to compare the safety and efficacy of a haemostatic matrix sealant agent, FloSeal with an infrared-sapphire coagulator (ISC), during open enucleoresection of renal cell carcinoma (RCC), to minimize or avoid suturing and warm ischemia time.

## Methods

From January 2006 and June 2009 all patients affected by a RCC were considered for this study. Of these, we enrolled only who has been selected to undergo a lumbar renal enucleoresection. Criteria required for performing an enucleoresection were a peripheral RCC with a diameter less or equal to 4 cm (stage T1a) [11]. Enucleoresection means to remove the tumor and its pseudocapsule with a normal renal parenchyma margin. With a blunt dissection by using monopolar electrocautery, the capsule of the tumour was incised circular about 5 mm around and the mass removed. The same surgeon performed all operations by a lumbar access.

Of 38 eligible patients, 8 declined to participate in the study and a total of 30 subjects were enrolled. The research has been carried out in accordance with the Declaration of Helsinki and approved by the Ethics Committee of our hospital. Consent was obtained from all patients after full explanation of the procedure. Patients were randomly assigned to one of the two haemostatic approaches: 15 (Group 1) to FloSeal (5,000 U/5 mL) (Baxter Inc, Deerfield, IL) and 15 (Group 2) to ISC (Saphir-Koagulator ISK 250, NK-OPTIK, München). Randomization number was assigned by using a random allocation software.

Before surgical treatment, subjects were evaluated with a detailed history, physical examination, standard blood chemical analyses, upper urinary tract and bladder ultrasound, abdomen computerized tomography without and with intravenous contrast medium.

FloSeal is composed of a bovine-derived gelatin matrix component and of a human-derived thrombin component; it works on wet, actively bleeding tissue. After identifying the source of bleeding at the tissue surface, manually a gauze sponge is approximated against the bleeding surface and with the applicator tip Floseal is applied between the sponge and the bleeding surface to create a small hill at the source of bleeding. The gauze sponge holds Floseal in place, against the bleeding surface. To minimize disruption of the clot, the gauze sponges is removed after hemostasis has been achieved.

ISC works through the conversion of light into thermal energy upon absorption by the bleeding tissue causes coagulation and haemostasis. By the ISC, light (wave length: 0.4-3 μm, power: 120W) from a halogen lamp is being transmitted to the bleeding tissue via a sapphire crystal, which is non-adhesive and of high thermal resistance. ISC is focused against the bleeding surface as long is necessary to achieve haemostasis.

The study endpoints for the evaluation of haemostatic efficacy were the rate of successful intra-operative haemostasis (identified by cessation of bleeding) and time required for haemostasis, overall post-operative bleeding, rate of transfusion, rate of surgical revision for bleeding and post-operative morbidity, were evaluated. The outcome measures were the patient's operative time, blood loss, intra-operative and post-operative complications, and length of hospitalization.

SPSS for Windows (version 10.0.7) computer package was used for statistical analysis. In order to detect a difference of 30% between the 2 groups in the effect size (two-side type I error of 5% and type II error of 0.2%) 30 patients were necessary. Statistical significance was accepted if p < 0.05. All statistical tests were two-tailed. As variables were not normally distributed (Shapiro-Wilk test; P < 0.05) continuous variables were analyzed with Wilcoxon-Mann Whitney Rank Sum Test. Categorical variables between groups were compared with Chi-square test or with Fisher's exact test when requested.

## Results

17 men and 13 women with a median age of 52.5 years (Group 1 vs 2; p > 0.05), were enrolled. In Table 1 we report the demographic characteristics and preoperative parameters of patients enrolled. As showed in the computerized tomography, the depth of penetration of tumors ranged from 1 to 3 cm (median 1,9 cm), without a direct contact with the upper excretory urinary tract (renal pelvis) in each of the patients enrolled. The tumor diameter ranged from 2.0 to 4.0 cm (median 3.3 cm), in both groups (p > 0.05). Renal hilar clamping was not required. Excision was performed with a monopolar electrocautery, and final haemostasis was obtained with FloSeal (Group 1) or with ISC (Group 2), without suturing. Statistically higher rates of successful haemostasis and shorter time-to-haemostasis (8,1 vs 12,9 min) were observed in the FloSeal group (p < 0.001 both) (Table 2). Patients operative time was not different between Group 1 vs 2 (58.7 vs 62.4; p > 0.05). The average blood loss during surgery was less for the FloSeal group than for the ISC group (60 vs 85 mL; p < 0.05). Postoperative median blood loss through the Jackson-Pratt drain was 25 mL for the FloSeal group and 46 mL for the control group (p < 0.05). In addition, wound drain removal occurred earlier, the day after surgery, with FloSeal (p = 0.04 vs. group 2). Transfusion of blood products and revision for bleeding were not required. Median length of the postoperative hospital discharge was 2.5 days for FloSeal group and 3.5 for the Group 2 (p < 0.05). Patients' discharge and removal of drains has been decided by the clinicians blinded to the treatment allocation.

**Table 1:** Demographic and pre-operative parameters of patients enrolled

	Group 1 (15 patients)	Group 2 (15 patients)	P values
Age (years)	51 (39-62)	53 (40-65)	0.26 ^2^

Gender n (%)	Male 8 (53%) Female 7 (47%)	Male 9 (60%) Female 6 (40%)	0.08 ^1^ 0.09 ^1^

Depth of penetration of tumors in renal parenchyma	1.9 (0.8-3.0)	1.9 (1.0-2.9)	0.90 ^2^

Tumor diameter	3.3 (2.4-4.1)	3.3 (2.1-4.5)	0.94 ^2^

^1^χ^2 ^corrected test or Fisher's Exact test; ^2 ^Wilcoxon-Mann Whitney Rank Sum Test.

**Table 2:** Intra-operative and post-operative results

	Group 1	Group 2	P values
Operative time (min)	58.7 (46.7-70.7)	62.4 (47.4-77.4)	>0.05 ^2^

Time to haemostaisis (min)	8,1 (7-9.1)	12,9 (10.7-15)	<0.001 ^2^

Intraoperative blood loss (mL)	60 (34.5-85.3)	85 (44.3-125.2)	<0.05 ^2^

Postoperative blood loss (mL)	25 (23.3-50.1)	46 (35.3-90.2)	<0.05 ^2^

Drain removal (days)	1 (1-2)	3 (1-4)	0.04 ^2^

Hospital discharge (days)	3 (2-4)	4 (4-6)	<0.05 ^2^

^1^χ^2 ^corrected test or Fisher's Exact test; ^2 ^Wilcoxon-Mann Whitney Rank Sum Test.

No major immediate or delayed complications were observed in either Groups. Pathology revealed a 90% of clear cell RCC, 8% of papillary and 2% of chromophobe RCC (Group 1 vs 2; p > 0.05). All margins of resection were negative. At a mean follow-up of 15 months (6-37 months) no recurrence was observed. Among all patients, the mean preoperative serum creatinine was 0.9 mg/dL, and the average level at a mean of 12 months postoperatively was 1.0 mg/dL (Group 1 vs 2; p > 0.05).

## Discussion

Topical HAs are classified by the FDA as absorbable or non-absorbable, or as sealants (liquid adhesives) and dressing (solid matrix) [12]. As it should be hard to conduct clinical trials on the use of HAs in several different surgical fields due to the individual variability of patients, there are not defined indications as regard their efficacy and safety and about how to choose the appropriate haemostatic. Several studies have been carried on to investigate the haemostatic capacity and stability of different HAs like gelatin (sponge and matrix), bovine thrombin, freeze-dried recombinant factor VIIa and microporous polysaccharide hemispheres, in experimental traumatic bleeding models) [13]. The reduction in blood loss after liver injury and in a grade 5 renal injury, with no delayed bleeding and nephrotoxicity, suggests a possible employ for FloSeal in the treatment of devastating renal injuries [14,15]. Furthermore, a porcine model investigating on the use of FloSeal and Tisseel in vascular and collecting-system injury during partial nephrectomy has showed that Tisseel alone is not adequate for either haemostasis or management of major collecting-system injury, while Floseal appears sufficient to control major vascular and collecting-system injuries [16]. All those studies paved the way for a large use of the aforementioned sealants in several surgical fields. In the urologic surgery, Floseal has been used for tubeless percutaneous nephrolithotomy in patients rendered completely stone free, administrating the haemostatic gelatin matrix to the nephrostomy tract, achieving immediate haemostasis and avoiding the placement of a nephrostomy tube [17,18]. With increasing surgical skills and novel methods of haemostasis, laparoscopic PN (LPN) has become an attractive treatment option for selected renal tumors [19,20]. In performing LPN, FloSeal and BioGlue have been proposed to avoid surgical bolsters or parenchymal sutures and to make more surgeons comfortable with the intricacies of laparoscopic suturing [21,22]. A recent survey on the current practice for urologists performing LPN, confirmed that HAs and/or glues were used in 77.4% of cases and were mainly represented by gelatin matrix thrombin (FloSeal), fibrin gel (Tisseel), bovine serum albumin (BioGlue), cyanoacrylate glue (Glubran), oxidized regenerated cellulose (Surgicel), or combinations of these. The overall postoperative bleeding requiring transfusion and urine leakage rates were 2.7% and 1.9%, respectively [23-29].

The widespread use of modern radiological techniques substantially changed clinical presentation of RCC in the last decades and, more than one half of all patients with surgically localized renal tumors are detected incidentally. All this lead to an increased interest in nephron-sparing surgery (NSS) for the treatment of small tumors. Enucleoresection is one of the NSS techniques available for the elective treatment of small RCC in stage T1a, allowing long-term cancer specific survival rates without an increased risk of local recurrence. We have chosen to perform the enucleoresection in a selected group of patients comparing two ways of achieving haemeostasis by FloSeal and ISC, in the attempt to verify differences related to time to haemostasis, blood loss, operative time and to spare or avoid renal parenchymal damage by heat and suturing. Our results were in accord with those reported in the current literature [30]. By applying Floseal during open enucleoresection of RCC, bleeding was efficiently controlled in all patients treated and none required post-operative transfusions or showed significant post-operative blood loss. Mostly important we compared the haemostatic efficiency of Floseal with the unusual application in the kidney surgery of ISC. ISC has been developed for the haemostasis of parenchymatous haemorrhage, mainly liver and spleen, showing that the time until haemostasis takes place was reduced 60% and depth of necrosis 25% in comparison to usual diathermia. Also by using the ISC, we achieved good results in term of bleeding control even if with longer time to haemostasis respect to FloSeal and a greater intra-operative blood loss. HAs like Floseal, used in the enucleresection of RCC, offer a good haemostatic control without the need of suturing. This suggestion is supported by our experience as we reached a satisfying haemostasis by applying the HA all over the wound ground or by using the infrared light on the bleeding sites.

Based on more recent data, FloSeal has been experimented in the robotic laparoscopic radical prostatectomy (RP) in the attempt to develop techniques cautery-free, clip-free and nerve-sparing that preserve the neurovascular bundles and minimize trauma, even if the effect on potency still needs further follow-up [31,32].

As the indications for topical HAs increase in urology, the question arises about what happens to these agents when they enter the urinary collecting system. It has been shown that fibrin glue and oxidized regenerated cellulose maintain a solid form when initially placed in direct contact with urine and then assume a semisolid gelatinous state. Polyethylene glycol forms a solid clot initially and does not change after 5 days. Only hemostatic gelatin matrix remained as a fine particulate suspension in both normal and sanguineous urine so that the implications of these findings with regard to sealing the renal parenchyma or the collecting system are still to be evaluated [33]. Nonetheless, nowadays the haemostatic agents are still expensive so that it has been proposed their use in major surgical procedures and in acute life-threatening hemorrhages or otherwise in moderately critical patients with severe concomitant diseases or coagulation disorders. The cost of the kit of Floseal is in US 85 dollars. Differently from Floseal the ISC is an equipment which, although more expensive at the beginning, allow to treat several patients even if with longer time to haemostasis according to our results. Also adverse events have been reported in several fields as consequence of the use of gelatin-thrombin. HAs may elicit a foreign body reaction leading to large giant cell granuloma, mimicking a metastatic disease [34,35]. HAs, frequently used during abdominal surgery, are linked to adhesion formation: a case of early post-operative small bowel obstruction during laparoscopic staging for endometrial cancer has been described, underling that the use of haemostatic agents should be considered as a cause in the differential diagnosis in patients with early post-operative bowel obstruction [36-38]. Not the least, application of FloSeal in the lumpectomy cavity has resulted in benign mammographyc microcalcifications that could be misinterpreted as malignant [39]. Therefore, it is crucial to get a better understanding of the genesis of those reactions.

Ahead of its time are, on one hand, the recently applications of HAs in the premature rupture of membranes where the adhesive sealants confer mechanical support to the membrane and form a water tight seal [40]. On the other hand, in the prostate cancer research frontiers, has been evaluated the potential use of intra-operative gelatin matrix haemostatic sealant embedded with macrophages transduced with murine interleukin 12 recombinant adenoviral vector for prevention of recurrence of prostate cancer following RP [41].

To our knowledge this is the first study which compare the use of a HA and of the ISC in the enucleoresection of RCC. The recent evidence related to the NSS capable, in selected cases, to offer the same results of partial and radical nephrectomy as regard the overall cancer-specific survival and the progression free survival, open the way to look for minimally invasive approach without the necessity of renal hilar clamping, avoiding the risks related to warm ischemia time. Bleeding of the kidney wound is usually controlled by bipolar coagulation and by suturing, potentially increasing the induced tissue damage by heat and by sutures. According with our results, it should be easily achieved, in selected patients with RCC of limited dimension, by using topic HAs or the ISC. Overall, in our procedure also the need of suturing was not required minimizing the operative time without any risk of further intra- or post- operative bleeding. Even if, it has been reported the use of thrombin-gelatin haemostatic matrix for both oozing and focal hemorrhage and effective even for high-flow bleeding, according with our experience, we suggest to apply HAs to control localized bleeding, for tumors of 7 cm or less of diameter. We propose to make use of those different way of achieving haemostasis considering the satisfying results, their handling and their efficacy, in the enucleoresecton of RCC looking to a further employ also in the nerve sparing RRP. Nevertheless, those haemostatics should also result in more patients, suffering from several comorbidities with consequence coagulation disorders, being able to undergo minimally invasive NSS. Even if we agree that HAs is the way to go, they are not free from adverse reactions such as the creation of inflammation and necrosis with the risk of adhesions formation, whose genesis need to be clarified and which claim further studies to improve the design of these agents in future. Moreover, additional investigations will clarify the indications and the best HAs to choose.

Looking to the future, minimally invasive surgery will further drive the use of topical HA in the urologic field. They offer promising employ in the laparoscopic and robotic surgery to avoid the intricacy of the laparoscopic sutures, to achieve rapidly hemostat in life-risk hemorrhages in complicate situations like during wars or in patients with an altered coagulation status.

## Conclusions

Our results show Floseal and ISC to be both safe and efficacy in achieving haemostasis in the enucleoresection of RCC in T1a stage, with a less intra-operative and post-operative blood loss as well as a shorter time to haemostasis of Floseal in respect to ISC.

## Competing interests

The authors declare that they have no competing interests.

## Authors' contributions

GP and CV have made contribution to conception and design, drafting and revising the manuscript, PS has acquired data and revising manuscript. All authors have contributed to analyse, interpret and approved the version to be published.
